# Tumorigenic hybrids between mesenchymal stem cells and gastric cancer cells enhanced cancer proliferation, migration and stemness

**DOI:** 10.1186/s12885-015-1780-1

**Published:** 2015-10-24

**Authors:** Jianguo Xue, Yuan Zhu, Zixuan Sun, Runbi Ji, Xu Zhang, Wenrong Xu, Xiao Yuan, Bin Zhang, Yongmin Yan, Lei Yin, Huijuan Xu, Leilei Zhang, Wei Zhu, Hui Qian

**Affiliations:** 1 Jiangsu Key Laboratory of Medical Science and Laboratory Medicine, School of Medicine, Jiangsu University, Zhenjiang, Jiangsu, P. R. China; 2 The Affiliated Hospital, Jiangsu University, Zhenjiang, Jiangsu 212000 P. R. China

**Keywords:** Cell fusion, Mesenchymal stem cells, Gastric cancer, EMT, Cancer progression

## Abstract

**Background:**

Emerging evidence indicates that inappropriate cell-cell fusion might contribute to cancer progression. Similarly, mesenchymal stem cells (MSCs) can also fuse with other cells spontaneously and capable of adopting the phenotype of other cells. The aim of our study was to investigate the role of MSCs participated cell fusion in the tumorigenesis of gastric cancer.

**Methods:**

We fused human umbilical cord mesenchymal stem cells (hucMSCs) with gastric cancer cells *in vitro* by polyethylene glycol (PEG), the hybrid cells were sorted by flow cytometer. The growth and migration of hybrids were assessed by cell counting、cell colony formation and transwell assays. The proteins and genes related to epithelial- mesenchymal transition and stemness were tested by western blot、immunocytochemistry and real-time RT-PCR. The expression of CD44 and CD133 was examined by immunocytochemistry and flow cytometry. The xenograft assay was used to evaluation the tumorigenesis of the hybrids.

**Results:**

The obtained hybrids exhibited epithelial- mesenchymal transition (EMT) change with down-regulation of E-cadherin and up-regulation of Vimentin, N-cadherin, α-smooth muscle actin (α-SMA), and fibroblast activation protein (FAP). The hybrids also increased expression of stemness factors Oct4, Nanog, Sox2 and Lin28. The expression of CD44 and CD133 on hybrid cells was stronger than parental gastric cancer cells. Moreover, the migration and proliferation of heterotypic hybrids were enhanced. In addition, the heterotypic hybrids promoted the growth abilities of gastric xenograft tumor *in vivo*.

**Conclusions:**

Taken together, our results suggest that cell fusion between hucMSCs and gastric cancer cells could contribute to tumorigenic hybrids with EMT and stem cell-like properties, which may provide a flexible tool for investigating the roles of MSCs in gastric cancer.

**Electronic supplementary material:**

The online version of this article (doi:10.1186/s12885-015-1780-1) contains supplementary material, which is available to authorized users.

## Background

Mesenchymal stem cells (MSCs) represent a subset of non-hematopoietic adult stem cells, which exhibit the potential to differentiate to diverse lineages, such as bone, adipose and cartilage tissues [1, 2]. In addition to the roles in tissue repair and regeneration, MSCs have been suggested as a critical component in tumor microenvironment, in which the soluble factors produced by inflammatory and tumor cells will recruit MSCs to the tumor sites [3]. Our previous study has demonstrated that bone marrow derived MSCs are recruited to the site of growing tumors and promote tumor growth in mouse xenograft models [4–6], suggesting that the interaction between MSCs and tumor cells is critical for tumor progression. However, the underlying mechanism remains unclear.

Cell fusion, a complex and highly regulated process in which two or more cells become one by merging their plasma membranes, plays critical roles in several physiological (fertilization, tissue regeneration) and pathophysiological (viral infection, cancer) events [7]. More and more findings have proposed that cell fusion may be involved in tumor progression [8–12]. The hybrids of cell fusion can be more malignant than their parental cells and possess enhanced ability to metastasize [13–15]. A model of “wolf in sheep’s clothing” is proposed to explain the link between cell fusion and metastasis. This model suggests that tumor cells become metastatic by fusion with normal cells that travel throughout the body freely [16]. For instance, tumor associated macrophage may fuse with epithelial cancer cells at the sites of primary tumor, giving rise to hybrids that have enhanced migratory and invasive capabilities [17].

MSCs are considered as one of the pivotal elements in the tumor microenvironment as well as a promising fusogenic candidate [18]. So, whether MSCs could merge with other cells, pre-malignant cells or cancer cells, and play an important role in the occurrence of tumor. A stem cell fusion model has emerged as a classical mechanism for tumor development. This model suggests that a fusion event between bone marrow-derived stem cell (BMDSC) and pre-malignant cells give rise to cancer [19]. Also, MSCs can fuse with different cancer cells spontaneously at low frequency. Several studies have shown that the hybrids between pre-malignant cell and stem cells are more malignant than the parental cells and gain self-renewal and migratory abilities, which highlight the pro-tumor role of stem cells by fusing with other cells [20–23].

Gastric cancer is the fourth most common cancer and the second leading cause of cancer-related death worldwide [24]. In our previous studies, we found that after treatment with gastric cancer cell-derived exosomes, hucMSCs differentiated into carcinoma-associated fibroblasts (CAFs) [25]. We have also previously reported that hucMSCs activated by macrophages promote both gastric epithelial cells and gastric cancer cells proliferation and migration [26]. However, few researches have been done into the effect of cell fusion of MSCs with gastric cancer cells on gastric carcinoma. In the present study we fused hucMSCs with gastric cancer cells and investigated the effect of fusion with hucMSCs on the biological properties of gastric cancer cells. We found that the hybrids of mesenchymal stem cells and gastric cancer cells contributed to highly malignant both with EMT and stem-cell like properties.

## Methods

### Ethics statement

Ethical and methodological aspects of the investigation protocols were approved by the ethical committee of Jiangsu University (2012258).

### Cell culture

Human gastric cancer cell lines HGC-27 and SGC-7901 were purchased from Cell Bank,Type Culture Collection Committee,Chinese Academy of Sciences (Shanghai, China). HGC-27 cells and SGC-7901 cells were maintained in high-glucose DMEM (H-DMEM, Life technologies, USA) with 10 % FBS. HucMSCs were obtained and identified as previously described [27]. HucMSCs were maintained in low-glucose DMEM (L-DMEM, Life technologies) with 10 % FBS. Cells were all incubated at 37 °C in humidified cell culture incubator with 5 % CO_2_ and the medium was changed every 3 days after the initial plating.

### Cell fusion and sorting

Gastric cancer cells (HGC-27 or SGC-7901) and hucMSCs were labeled with DIO and DID fluorescent dye following the manufacturer’s instructions (Life technologies), respectively. The hybrids of DIO-labeled gastric cancer cells (1 × 10^6^) and DID-labeled hucMSCs (1 × 10^5^) were generated by using PEG1500 (Roche, USA). The fusion cells were plated in L-DMEM with 10 % FBS, cultured for 2 days, and then sorted by flow cytometer (SORP Aria II, BD Biosciences, USA). The double-positive hybrid cells were collected in L-DMEM containing 10 % FBS, penicillin and streptomycin. The sorted fused cells were collected and cultured in a 96-well plate using limiting dilution method for single cell sub-cloning.

### Flow cytometry and imaging

The DIO-labeled HGC-27 cells/SGC-7901 cells and DID-labeled hucMSCs was fused by PEG1500 *in vitro* and suspended in 200 μl PBS. Then the cell suspensions were analyzed on the Image Stream ^X^ Mark IIimaging flow cytometer (Merck Millipore) with low flow rate/high sensitivity. The cell suspensions were acquired immediately and single cell populations were gated for detect the fused cells and unfused cells visually. Four fluorescence channels were visualized in the INSPIRE software: Brightfield images were collected in CH1, DIO fluorescence was recorded using excitation with a 488 nm laser (CH2), and DID fluorescence using excitation with a 640 laser (CH11). A total of 3000–5000 cell events were collected for each sample. Single stained controls were also collected (DIO only and DID only labelled cells) at the same settings in order to develop a compensation matrix for removing spectral overlap of dyes from each of the channels.

### Cell counting

The parental and fusion cells were seeded into 24-well plate (1 × 10^4^ cells/well) overnight. The cells were collected and counted at the indicated time points (24, 48, 72 and 96 h). The results are the mean values of three independent experiments.

### Colony forming assay

The parental or fusion cells were harvested and plated into a 6-well plate (2 × 10^3^ cells/well) and incubated at 37 °C in humidified cell culture incubator with 5 % CO_2_ for 15 days. The medium was changed every 3 days. To evaluate the number of colonies, the cultures were fixed with 4 % para-formaldehyde and stained with crystal violet. The results are the mean values of three independent experiments.

### Cell invasion and migration

The parental or fusion cells (1 × 10^5^ cells in serum free-DMEM medium) were seeded into the upper chamber, and medium containing 10 % FBS was added to the lower chamber. After incubation at 37 °C in 5 % CO_2_ for 12 h, the cells that invaded and migrated to the lower surface of the membrane were fixed with 4 % para-formaldehyde and stained with crystal violet for 15 min. This experiment was performed in triplicate.

### Western blot

Cells were homogenized and lysed in RIPA buffer supplemented with proteinase inhibitor. Equal amount of proteins (150 μg) were loaded and run on 12 % SDS-PAGE gel, then transferred onto PVDF membranes following electrophoresis. After blocked with 5 % milk in TBS/T for 1 h, membranes were incubated with the primary antibodies at 4 °C overnight. The sources of primary antibodies were: anti-E-cadherin and anti-N-cadherin (Santa Cruz Biotechnology, CA, USA); anti-Oct4, anti-Sox2, anti-Nanog, anti-Vimentin (Signalway Antibody, USA); anti-PCNA, anti-Cyclin D1 (Bioworld Technology, Louis Park, MN, USA). GAPDH (Cwbio, Beijing, China) was used as the loading control.

### Real-time RT-PCR

Total RNA was extracted using Trizol reagent (Life technologies, Carlsbad, CA, USA) according to the manufacturer’s instructions and equal amount of RNA was used for real-time PCR analyses. The cDNAs were synthesized by using a reverse transcription kit (Vazyme, Nanjing, China). β-actin was used as the internal control. The sequences of specific primers are listed in Table 1.

**Table 1:** List of primer sequences

Genes name	Forward primer	Tm(°C)	Length(bp)
	Reverse primer		
Oct4	TTGAGGCTCTGCAGCTTAG	60	285
	GCCGGTTACAGAACCACAC		
Sox2	ACACCAATCCCATCCACACT	60	224
	GCAAACTTCCTGCAAAGCTC		
Nanog	CCTGATTCTTCCACCAGTCC	60	292
	TGCTATTCTTCGGCCAGTTG		
Lin28	ACCGGACCTGGTGGAGTATT	60	204
	CTTCAGCGGACATGAGGCTA		
E-cadherin	CGCATTGCCACATACACTCT	60	252
	TTGGCTGAGGATGGTGTAAG		
N-cadherin	AGTCAACTGCAACCGTGTCT	60	337
	AGCGTTCCTGTTCCACTCAT		
vimentin	GAGCTGCAGGAGCTGAATG	60	344
	AGGTCAAGACGTGCCAGAG		
α -SMA	CTGACTGAGCGTGGCTATTC	58	452
	CCACCGATCCAGACAGAGTA		
FAP	ATAGCAGTGGCTCCAGTCTC	59	278
	GATAAGCCGTGGTTCTGGTC		
Slug	CCTGGTTGCTTCAAGGACAC	60	395
	TCCATGCTCTTGCAGCTCTC		
snail	GGTTCTTCTGCGCTACTGCT	59	285
	TAGGGCTGCTGGAAGGTAAA		
twist	GTCCGCAGTCTTACGAGGAG	60	294
	TGGAGGACCTGGTAGAGGAA		
β-actin	CACGAAACTACCTTCAACTCC	56	265
	CATACTCCTGCTTGCTGATC		

### Immunofluorescence

Cells cultured in 24-well chamber slides were washed twice with cold PBS, fixed with 4 % para-formaldehyde for 15 min, permeabilized with 0.1 % Triton X-100 for 5 min, blocked with 5 % BSA, incubated with indicated primary antibodies(anti-CD44 and anti-α-SMA, Bioworld Technology) at 4 °C overnight and followed by a Cy3-conjugated anti-rabbit secondary antibody (Cwbio, Beijing, China). The cells were then stained with Hoechst 33342 for nuclear staining, and the images were acquired with a Nikon eclipse Ti-S microscope (Nikon, Tokyo, Japan).

#### Flow cytometry

The expression of CD133 antigen on hybrids and parental gastric cancer cells were performed by flow cytometry. Cells were stained with PE-conjugated monoclonal anti-human CD133 (Becton Dickinson). Isotype control IgG-PE (San Jose, CA) served as a control. After stained 30 min, samples were analyzed by flow cytometry (FACS Calibur, BD) and data were analyzed using CellQuest software (BD Biosciences).

#### H&E staining

The neoplasm tissues (4 mm2) were deparaffinated then gradually dehydrated, embedded in paraffin, the tissue sections (4 μm) were stained by H&E staining for light microscopy.

### Xenograft assay

Twelve male BALB/C nude mice (4–6 weeks) were purchased from Laboratory Animal center of Shanghai and were randomly divided into 6 mice per group. Both groups were injected subcutaneously of either HGC-27 or HGC-27 fusion cells (2 × 10^6^ cells in 200 μl PBS). Tumor growth was evaluated by measuring the length and width of the tumor mass with calipers every 2 days. Tumor volumes were calculated by the modified ellipsoidal formula: (length × width2) /2.

### Statistical analysis

Statistical analysis of the data was performed by using GraphPad Prism 5 software. All the data were expressed as mean ± SD. The means of different treatment groups were compared by two-way ANOVA or the Student’s t test. *P* value <0.05 was considered statistically significant.

## Results

### Fusion of gastric cancer cells with hucMSCs generates hybrid cells

**Fig. 1:**
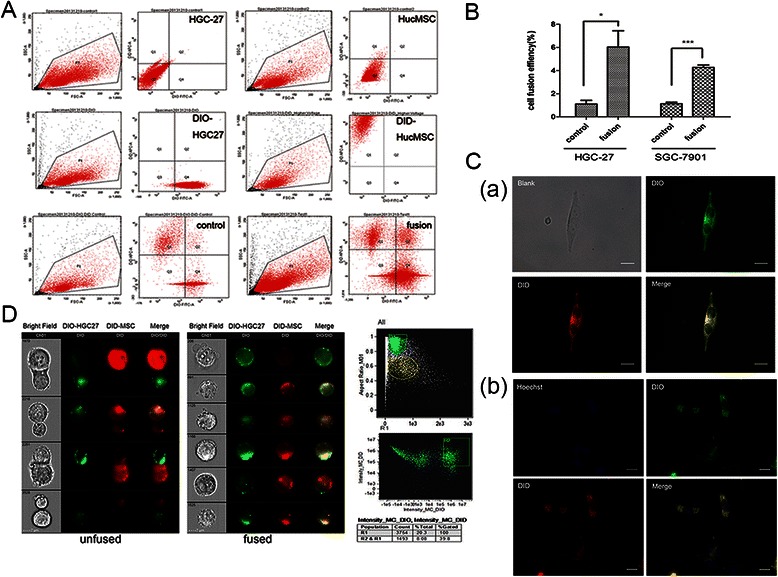
Cell fusion between hucMSCs and gastric cancer cells. **a**. Cell sorting of HGC27-hucMSC fused cells by flow cytometry. HGC-27 and hucMSCs were labeled with DIO and DID, respectively. DIO-HGC27 and DID-hucMSC was collected respectively. The control group indicates spontaneous fusion while the fusion represents the PEG1500-mediated generation of double positive hybrids. **b**. The statistical analyses of fusion efficiency. The data represent mean ± SD of three independent experiments. *P < 0.05,***P < 0.001 (**c**) Microscopic fluorescent images of fused cells. (*a*) The single double-positive hybrid was detected on the third day after sorting. Scale bar 100 μm, Magnification: ×200; (*b*) Hybrids with two nuclei were stained with Hoechst 33342 (blue) and cell membrane structures were double-labeled (yellow) were observed on the seventh day after sorting. Scale bar 100 μm, Magnification: ×200. **d**. Representative images from the cell population gates were tested by imaging cytometer. HGC-27 and hucMSCs were stained with DIO (green) and DID (red), respectively. In the “unfused”group, under the treatment of PEG1500, the two cells formed an adhesion structure but not a hybrid. Also, the hybrids fused with DIO-HGC27 and DID-HucMSCs are yellow and showed in the“fused” group. The double positive cell populations were gated in R2, the population of hybrids was 8.08 %

To facilitate the identification of cell fusion events, fusion partners were labeled with cytomembrane fluorescent dyes DIO (for HGC-27 and SGC-7901 cells) and DID (for hucMSCs). Cell fusion was induced by PEG1500 in co-cultured DIO-HGC27 and DID-hucMSCs. After culture for 24 h *in vitro*, the hybrids (double-stained cells) were detected and sorted by dual color (DIO and DID) fluorescence activated cell sorting (FACS) for initial enrichment of the hybrid cells (Fig. 1a). sThe cell sorting experiment of DIO-SGC7901 and DID-hucMSCs was also tested (Additional file 1). The fusion efficiency was about 6.033 ± 1.408 % in HGC-27 cells and 4.067 ± 0.033 % in SGC-7901 cells (Fig. 1b). The statistical data was listed in Table 2. The sorted fused cells were collected and cultured 96-well plate using limiting dilution method for single cell sub-cloning. After culture for 3 days, the dual fluorescent binucleate hybrids were identified by confocal microscope (Fig. 1c). The cell fusion event and cell fusion efficiency was also demonstrated by imaging flow cytometer (Fig. 1d), which combines the digital fluorescence microscopy with speed and sensitivity of flow cytometry. The flow cytometry and imaging of cell fusion event between SGC-7901 and hucMSCs was showed (Additional file 2). The single double-positive hybrid in a well was selected and cultured for expansion. Then, we obtained the cell lines and named it HGC-27 fusion. The SGC-7901 fusion cell lines were obtained in the same way.

**Table 2:** Cell fusion efficiency of control and fusion group

	HGC-27(%)		SGC-7901(%)	
	control	fusion	control	fusion
1	1.7	4.2	1.2	4.0
2	1.0	5.1	1.3	4.1
3	0.6	8.8	0.9	4.1

### Cell fusion enhanced growth of gastric cancer cells

**Fig. 2:**
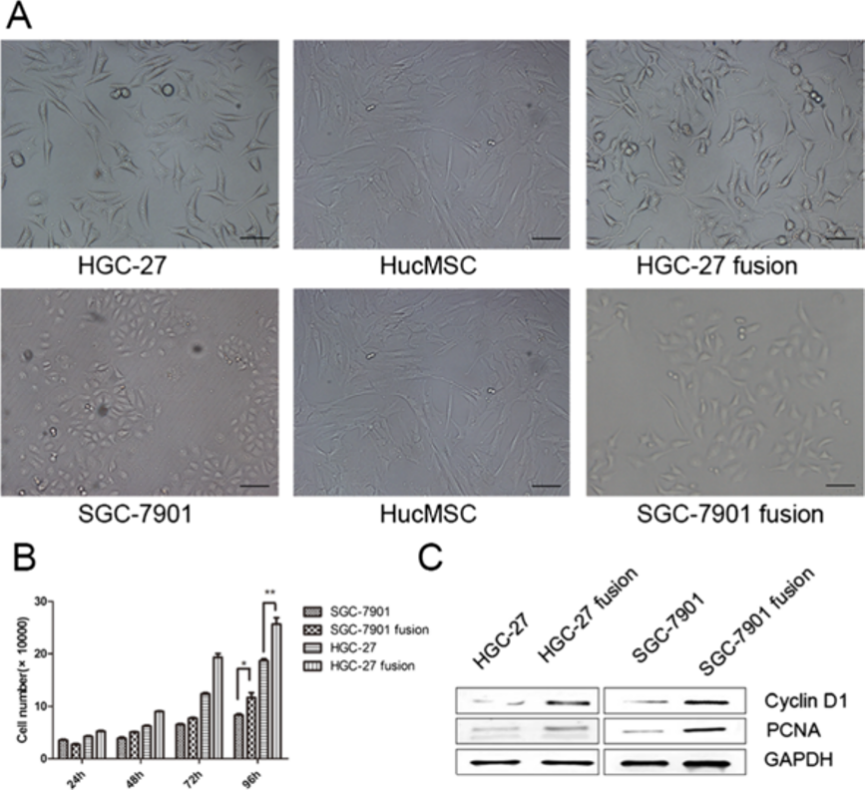
Fusion with hucMSCs induces morphological changes and enhanced growth of gastric cancer cells. **a**. The two gastric cancer cell lines showed epithelial morphology and hucMSCs with fibroblast-like shape. HGC-27 fusion hybrids exhibit elongated shape and front-to-back polarity. SGC-7901 fusion lost the epithelial morphology and assumed a fibroblast-like appearance. Scale bar 100 μm, Magnification: ×100. **b**. The growth of the parental and hybrid cells was determined by cell counting assay. **c**. The expression of PCNA and CyclinD1 proteins in parental and hybrid cells was examined by western blot

Morphological observation showed that the parental gastric cancer cells displayed rounded and elongated morphologies, respectively. After fusion with hucMSCs, the hybrid cells lost epithelial morphology, became scattered and exhibited a fibroblast-like appearance with an elongated shape and started to grow as bundles (Fig. 2a). To further investigate the effect of cell fusion on cell growth ability, we compared *in vitro* growth rates of the hybrid cells with that of their parental gastric cancere cells by cell counting assay. At the fourth day after cell seeding, the number of hybrid cells was markedly higher than that of their parental cells (Fig. 2b). The proliferating ability of the hybrid cells was determined by colony forming assay. Statistical results showed that the hybrid cells grew faster and formed more colonies than parental cells (3–4 folds) (Additional file 3). We also examined the expression of PCNA and cell cycle regulatory protein (CyclinD1) by western blot. We found that PCNA and CyclinD1 levels were significantly increased in fusion cells (Fig. 2c). In summary, these data suggest that cell fusion enhances the growth ability of gastric cancer cells *in vitro*.

### Cell fusion promoted EMT of gastric cancer cells

**Fig. 3:**
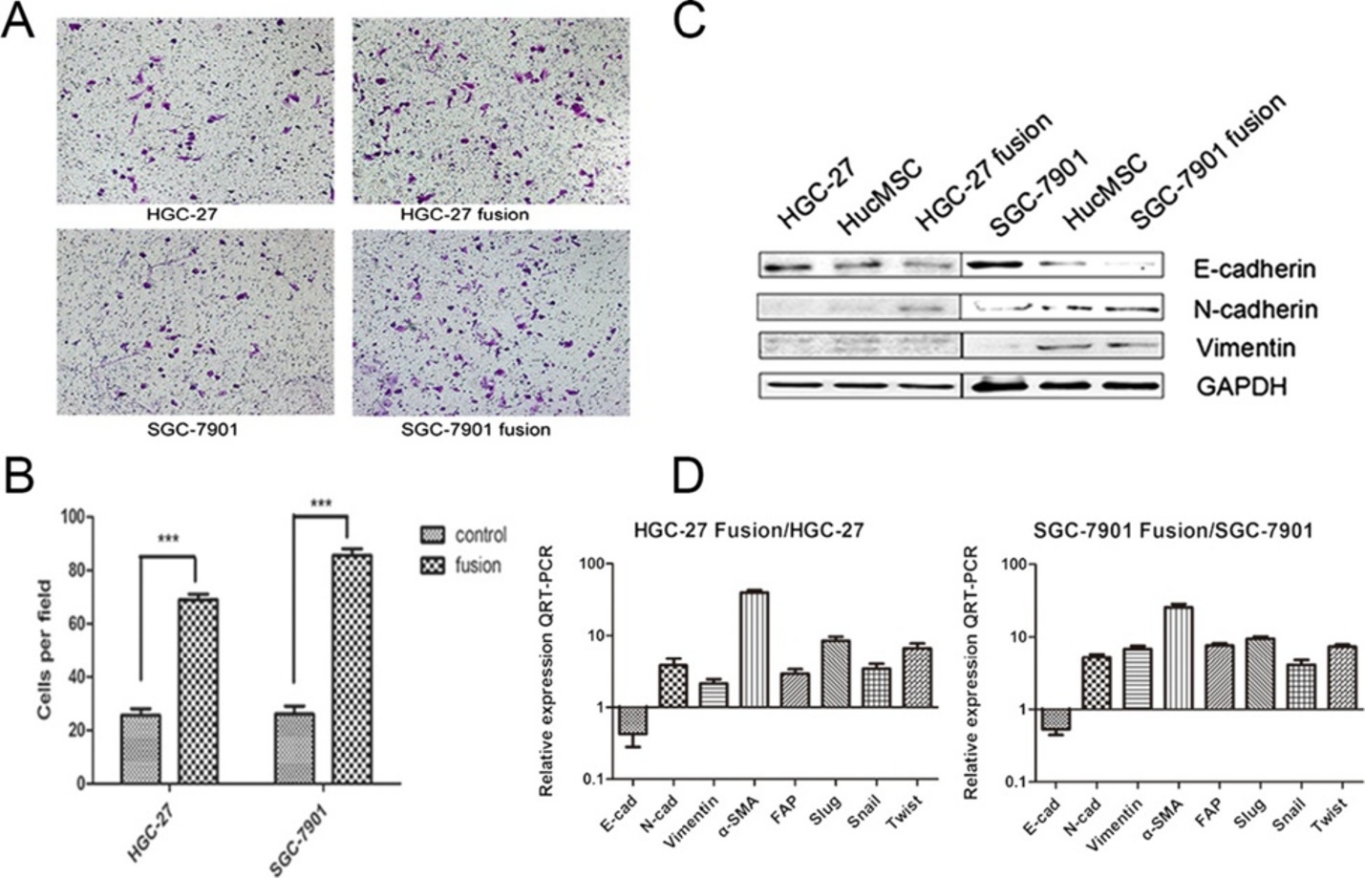
Fusion with hucMSCs induces EMT in gastric cancer cells. **a**. Transwell migration assay of parental and hybrid cells. **b**. The number of migrated cells in transwell migration assay. ***P < 0.001. **c**. The expression of mesenchymal markers E-cadherin、N-cadherin and Vimentin was determined by western blot. **d**. The expression of EMT-related genes was determined by real-time RT-PCR

Due to the morphological changes of MSC-gastric cancer cell hybrids, we hypothesized that the fused cells might undergo an epithelial to mesenchymal transition. To this end, transwell migration assay and wound healing assay was carried out to determine the migratory ability of the cell hybrids. In transwell migration assay, the number of HGC-27 fusion cells migrating through the transwell membrane was more migratory (3.2-fold) compared of HGC-27 cells. The number of SGC-7901 fusion cell hybrids migrating through the transwell membrane was more 4.1-fold than that of SGC-7901 cells (Fig. 3a and b). To investigate whether EMT-associated genes are differentially expressed between the fused cells and the parental cells, we used western blot and real-time RT-PCR to determine the expression of EMT related proteins and genes in fused cells and parental cells. The results of western blot revealed that the fused cells exhibited an obvious decrease in E-cadherin expression while increase in the expression of vimentin and N-cadherin compared to their parental gastric cancer cells (Fig. 3c). The results of real-time RT-PCR revealed that the level of E-cadherin mRNA in hybrids was obviously down-regulated but the expression of mesenchymal markers (α-SMA, FAP, vimentin, N-cadherin, snail, slug, and twist) in fusion cells were evidently increased (Fig. 3d). In summary, these data suggest that, after fusion with hucMSCs, the migratory ability of gastric cancer cells is changed markedly and indicates the hybrid cells experience the process of EMT.

### Cell fusion increased gastric cancer cell stemness

**Fig. 4:**
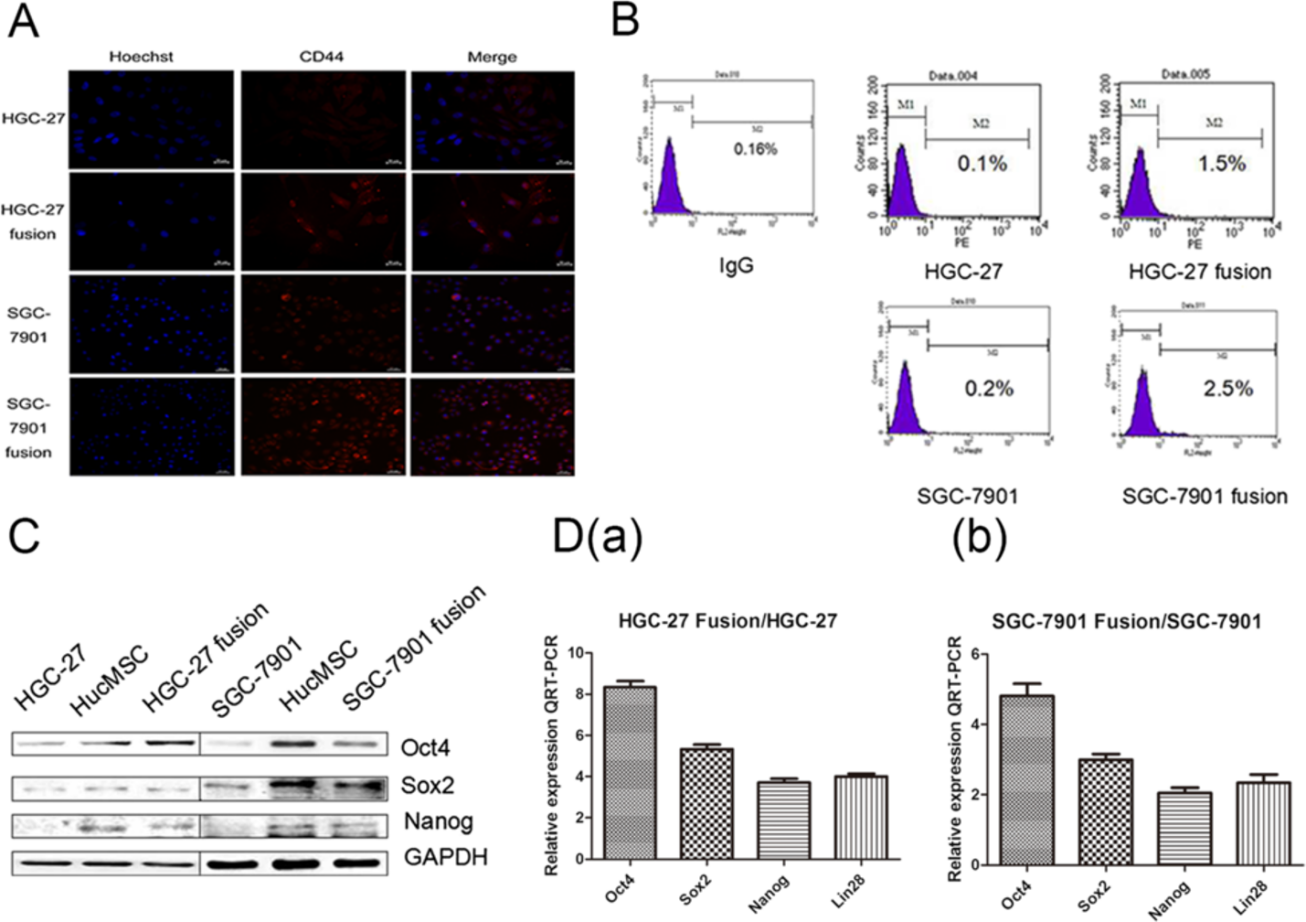
Fusion with hucMSCs induces the acquisition of stemness in gastric cancer cells. **a**. Immunofluorescent staining of CD44 in the parental and the hybrid cells. **b**. The expression of cancer stem cell marker CD133 in the parental and hybrid cells was determined by flow cytometry. **c**. The expression of Oct4, Sox2, Nanog proteins in the parental and hybrid cells was determined by western blot. **d**. The expression of Oct4, Sox2, Nanog, and Lin28 in the hybrid cells related to the parental cells was examined by real-time RT-PCR

Our present results demonstrate that the hybrid cells may have undergone an epithelial-mesenchymal transition,recent studies have demonstrated that EMT plays a key role in generating cancer stem cells. So, whether our hybrid cells could acquire stem cell properties? To validate it, we performed real-time RT-PCR, western blot to determine the stemness of the cell hybrids. First, we tested cancer stem cell surface marker CD44 by immunofluorescence. Obviously, the expression of CD44 on hybrid cells was stronger than the parental gastric cancer cells (Fig. 4a). CD133, a member of prominin family, was first discovered in hematopoietic stem cells, and served as one of the most widely reported marker of cancer stem cells. To determine whether the hybrid cells possess the cancer stem cell properties, we first assessed the expression of CD133 on hybrids and parental gastric cancer cells by flow cytometry. CD133 expression increased in hybrids compared with the parental cells (increase from 0.1 to 1.5 % in HGC-27 fusion and 0.2 to 2.5 % in SGC-7901 fusion) (Fig. 4b). The expression of stemness factors including Oct4, Sox2, Nanog and Lin28, which are known to be sufficient to reprogram somatic cells to pluripotent stem cells, were found to be significantly increased in fused cells compared to the parental gastric cancer cells by western blot and real-time RT-PCR (Fig. 4c and d). All these results indicate that the hybrid cells may acquire multiple traits of stem cells.

### Cell fusion enhanced growth of gastric cancer cells *in vivo*

**Fig. 5:**
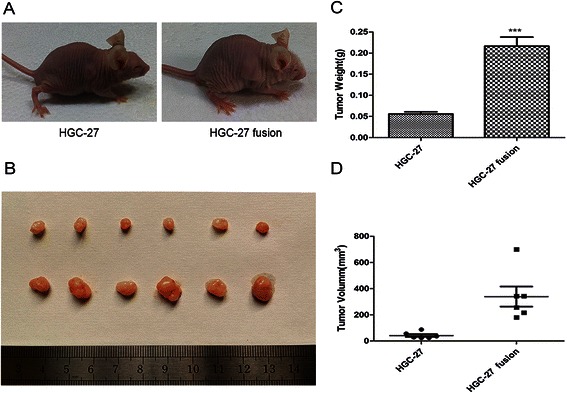
Hybrids of HGC-27 cell promote gastric cancer growth *in vivo*. **a**. The representative images of tumor-bearing mice. **b**. Tumor tissues were photographed 20 days after tumor cell inoculation. **c**, **d**. The weight and volume of tumor issues removed from HGC-27 and HGC-27 fusion groups. ***P < 0.0001

To investigate whether cell fusion of gastric cancers and hucMSCs could promotes cancer growth *in vivo*. We used HGC-27 cells and HGC-27 fusion cells to establish xenograft tumor models in nude mice. The images of tumor-bearing mice were shown (Fig. 5a). In the HGC-27 fusion group, tumor nodule started to form at the 4 days after injection while the HGC-27 cells group was not observed. As shown in (Fig. 5b, c and d), the tumor weight and volume in HGC-27 fusion group was higher than that in the HGC-27 group. The neoplasm tissues of hybrids presented highly heterogeneity, abnormally elevated nuclear/cytoplasmic ratios, and derangement distribution in some regions (Additional file 4). Taken together, these results suggest that cell fusion between gastric cancer cells and hucMSCs enhances gastric cancer growth *in vivo*.

## Discussion

During the process of tumor progression, tumor cells can detach from the primary tumor site and spread to other parts of the body and colonize distant organ sites. Although a number of routes to metastasize have been proposed, the precise underlying mechanisms still remain elusive. Recently, a prominent theory has emerged, which states that a tumor cell could fuse with a mobile cell type and then travel to another site in the body to establish cancer. This classic theory is called “cancer cell fusion” [28], which first regard cell fusion event as a possible mechanism of tumor metastasis. Pawelek et al. fused the healthy macrophages with weakly metastatic melanoma cells and found that most of the experimental hybrids were highly metastatic and lethal when implanted into mice [29]. Not only in animal model, they also give substantiated reports for cancer cell fusion in human, in which a melanoma brain metastasis with a donor-patient hybrid genome following bone marrow transplantation [30]. Thus, cell fusion event is regarded as a hidden force or a hidden enemy in cancer.

Similar to “cancer cell fusion” theory, another model has been proposed in recent years. The stem cell fusion model focuses on the role of BMDCs (including MSCs) in cell fusion event. This hypothesis proposes that fusion between BMDCs and “altered” tissue cells/pre-malignant cells would result in malignant tumors, which may be more migratory and more invasive. BM-MSC could be a putative fusion candidate and are known to specifically migrate to and engraft at inflammation or tumor sites. Several studies have reported that MSCs could fuse with variety of target cells and generate the tumorigenic hybrids after fusion. Houghton et al. reported that bone marrow derived cells are the origin of gastric cancer in helicobacter-infected mice [31]. MSCs may be recruited to the Helicobacter Pylori-infected gastric mucosa where they fuse with existing neoplastic and pre-neoplastic epithelial cells. Fusion of MSC with gastric epithelial cells increases invasion and metastasis of gastric cancer [32]. The spontaneous formation of BM-MSCs and lung/breast cancer hybrids acquire the tumorigenic and metastatic properties as well as mesenchymal characteristic [15, 22]. In addition, the hybrids of HepG2 cells and MSCs after PEG mediated fusion are more metastatic *in vivo* than MSCs and HepG2 [23]. In contrary, there are some studies showing that fusion of MSCs with esophageal carcinoma cells inhibits the tumorigenicity of esophageal carcinoma cells [33].

In this report, we fused human umbilical cord mesenchymal stem cells with gastric cancer cells by PEG1500 to obtain hybrids *in vitro*. PEG is a widely used agent for cell fusion because of its simplicity and low cost. Moreover, cell fusion mediated by PEG is an efficient procedure for obtaining somatic cell hybrids and widely used in monclonal antibody production. PEG could induce cell agglutination and cell-to-cell contact, leading to subsequent cell fusion. However, the detailed mechanisms underlying the PEG-mediated cell fusion are not known. In natural process, cell fusion is also a common event, compared with PEG-induced cell fusion, in natural process, cell fusion is a basic physiological activities and complex and highly regulated process, and the rate of cell fusion event is very rare. The aritifical fusion process such as PEG-induced cell fusion also has its limitation, PEG can cause the uncontrollable fusion of multiple cells, leading to the appearance of giant polykaryons. In addition, standard PEG-mediated cell fusion is pooly reproducible, and different cell types have varible fusion susceptibilities.

Our present results demonstrate that cell fusion between hucMSCs and gastric cancer cells generates a population of tumorigenic hybrids, which exhibit mesenchymal phenotypes and properties from MSCs along with increased metastatic capacity. The fused cells expressed higher levels of markers and regulatory proteins associated with epithelial to mesenchymal transition such as vimentin, α-SMA, and FAP, and exhibited an enhanced invasiveness and motility in transwell assay. This indicates that cell fusion event may induce an epithelial-mesenchymal transition (EMT) in the hybrids.

EMT is an essential step in the process of cancer cell dissemination and metastasis. Recent work reveals that the process of EMT generates cells with stem cell like properties in the mammary cell population [34], which puts forward the cell fusion hypothesis of cancer stem cells [35]. Fusion events may result in the transient induction of an EMT in large population of cancer cells and simultaneously induces the generation of CSCs. The cell fusion hypothesis of CSCs adds an important functional underpinning to the potential multifaceted roles of cell fusion in the initiation and progression of cancers [36, 37]. However, opinions differ as to whether cell fusion would generate CSCs. Fan et al. have shown that fusion between human bone hematopoietic stem cells and esophageal cancer cell might not contribute to the origin of cancer stem cells [38]. Our present data showed that the hybrids highly expressed stemness genes such as Oct4, Sox2, Nanog and Lin28 compared to the parental cells, indicating that the hybrids may acquire cancer stem cell properties after cell fusion. In our results, compared with the expression of EMT and stemenss proteins in gastric cancer cells and hucMSCs, the hybrid cells were somewhere in between. The results suggest that hybrid cells could acquire the mesenchymal and stemness proteins during a physical fusion event with MSCs. Although the hybrid cells have both EMT and stem cell-like properties, future work are warranted to ascertain whether the tumorigenic hybrids are cancer stem cells. Therefore, more attention should be paid to the cell fusion event in the cancer research in the future, especially the involvement of MSCs participated cell fusion in carcinogenesis. Blocking cell fusion within cancerous tissues would prevent the origin of more malignant tumor hybrid cells [39].

## Conclusions

The current results of our study demonstrated that cell fusion between hucMSCs and gastric cancer cells could give rise to a subpopulation of hybrid cells exhibiting an altered phenotype, including the morphological changes with a fibroblast-like appearance as well as the capability of both EMT and stem-cell like properties.

## Additional files

Additional file 1: **Flow cytometry analysis and sorting of hybrids. SGC-7901 cells were labeled with DIO, hucMSCs were labeled with DID.** The control group was the mock fused stained cells. The double positive fused cells were sorted and collected for the subsequent research. (TIFF 854 kb)
Additional file 2: **Representative images from each of the cell population gates.** DIO-labeled SGC-7901 showed green, DID-labeled hucMSCs showed red (upper panel). In the fusion cell populations, the unfused cells and fused cells that with double-positive and yellow color were displayed in the lower panel. (TIFF 1104 kb)
Additional file 3: **Representative images of cell colonies for HGC-27 and SGC-7901 parental and hybrid cells.** (A) The proliferating ability of the hybrid cells was determined by colony forming assay. (B) Statistical results showed that the hybrid cells grew faster and formed more colonies than parental cells (3–4 folds). (TIFF 1834 kb)
Additional file 4: **Histological images of tumor from a mouse injected with the HGC-27 cells and HGC-27 fusion cells. Magnification, ×400.** (TIFF 3256 kb)

## References

[1] Jiang Y, Jahagirdar BN, Reinhardt RL, Schwartz RE, Keene CD, Ortiz-Gonzalez XR (2002). Pluripotency of mesenchymal stem cells derived from adult marrow. Nature.

[2] Pittenger MF, Mackay AM, Beck SC, Jaiswal RK, Douglas R, Mosca JD (1999). Multilineage potential of adult human mesenchymal stem cells. Science.

[3] Gomes CM (2013). The dual role of mesenchymal stem cells in tumor progression. Stem Cell Res Ther.

[4] Zhu W, Xu W, Jiang R, Qian H, Chen M, Hu J, et al. Mesenchymal stem cells derived from bone marrow favor tumor cell growth *in vivo*. Exp Mol Pathol. 2006;80(3):267–74. doi:10.1016/j.yexmp.2005.07.004. 10.1016/j.yexmp.2005.07.004 16214129

[5] Zhu W, Huang L, Li Y, Zhang X, Gu J, Yan Y, et al. Exosomes derived from human bone marrow mesenchymal stem cells promote tumor growth *in vivo*. Cancer Lett. 2012;315(1):28–37. doi:10.1016/j.canlet.2011.10.002. 10.1016/j.canlet.2011.10.002 22055459

[6] Zhu W, Huang L, Li Y, Qian H, Shan X, Yan Y (2011). Mesenchymal stem cell-secreted soluble signaling molecules potentiate tumor growth. Cell Cycle.

[7] Ogle BM, Cascalho M, Platt JL (2005). Biological implications of cell fusion. Nat Rev Mol Cell Biol.

[8] Chakraborty AK, Sodi S, Rachkovsky M, Kolesnikova N, Platt JT, Bolognia JL (2000). A spontaneous murine melanoma lung metastasis comprised of host x tumor hybrids. Cancer Res.

[9] Lu X, Kang Y (2009). Cell fusion as a hidden force in tumor progression. Cancer Res.

[10] Lu X, Kang Y (2009). Efficient acquisition of dual metastasis organotropism to bone and lung through stable spontaneous fusion between MDA-MB-231 variants. Proc Natl Acad Sci U S A.

[11] Pawelek JM (2000). Tumour cell hybridization and metastasis revisited. Melanoma Res.

[12] Vignery A (2005). Macrophage fusion: the making of osteoclasts and giant cells. J Exp Med.

[13] Busund LT, Killie MK, Bartnes K, Seljelid R. Spontaneously formed tumorigenic hybrids of Meth A sarcoma cells and macrophages *in vivo*. Int J Cancer. 2003;106(2):153–9. doi:10.1002/ijc.11210. 10.1002/ijc.11210 12800188

[14] Ding J, Jin W, Chen C, Shao Z, Wu J (2012). Tumor associated macrophage x cancer cell hybrids may acquire cancer stem cell properties in breast cancer. PLoS One.

[15] Rappa G, Mercapide J, Lorico A (2012). Spontaneous formation of tumorigenic hybrids between breast cancer and multipotent stromal cells is a source of tumor heterogeneity. Am J Pathol.

[16] Duelli D, Lazebnik Y (2003). Cell fusion: a hidden enemy?. Cancer Cell.

[17] Clawson GA (2013). Cancer. Fusion for moving. Science.

[18] Wei HJ, Nickoloff JA, Chen WH, Liu HY, Lo WC, Chang YT (2014). FOXF1 mediates mesenchymal stem cell fusion-induced reprogramming of lung cancer cells. Oncotarget.

[19] He X, Tsang TC, Pipes BL, Ablin RJ, Harris DT (2005). A stem cell fusion model of carcinogenesis. J Exp Ther Oncol.

[20] Rizvi AZ, Swain JR, Davies PS, Bailey AS, Decker AD, Willenbring H (2006). Bone marrow-derived cells fuse with normal and transformed intestinal stem cells. Proc Natl Acad Sci U S A.

[21] Schichor C, Albrecht V, Korte B, Buchner A, Riesenberg R, Mysliwietz J, et al. Mesenchymal stem cells and glioma cells form a structural as well as a functional syncytium *in vitro*. Exp Neurol. 2012;234(1):208–19. doi:10.1016/j.expneurol.2011.12.033. 10.1016/j.expneurol.2011.12.033 22230665

[22] Xu MH, Gao X, Luo D, Zhou XD, Xiong W, Liu GX (2014). EMT and acquisition of stem cell-like properties are involved in spontaneous formation of tumorigenic hybrids between lung cancer and bone marrow-derived mesenchymal stem cells. PLoS One.

[23] Li H, Feng Z, Tsang TC, Tang T, Jia X, He X, et al. Fusion of HepG2 cells with mesenchymal stem cells increases cancerassociated and malignant properties: an *in vivo* metastasis model. Oncol Rep. 2014;32(2):539–47. doi:10.3892/or.2014.3264. 10.3892/or.2014.3264 24926698

[24] Brenner H, Rothenbacher D, Arndt V (2009). Epidemiology of stomach cancer. Methods Mol Biol.

[25] Gu J, Qian H, Shen L, Zhang X, Zhu W, Huang L (2012). Gastric cancer exosomes trigger differentiation of umbilical cord derived mesenchymal stem cells to carcinoma-associated fibroblasts through TGF-beta/Smad pathway. PLoS One.

[26] Yang T, Zhang X, Wang M, Zhang J, Huang F, Cai J (2014). Activation of mesenchymal stem cells by macrophages prompts human gastric cancer growth through NF-kappaB pathway. PLoS One.

[27] Qiao C, Xu W, Zhu W, Hu J, Qian H, Yin Q (2008). Human mesenchymal stem cells isolated from the umbilical cord. Cell Biol Int.

[28] Pawelek JM (2008). Cancer-cell fusion with migratory bone-marrow-derived cells as an explanation for metastasis: new therapeutic paradigms. Future Oncol.

[29] Pawelek JM, Chakraborty AK (2008). Fusion of tumour cells with bone marrow-derived cells: a unifying explanation for metastasis. Nat Rev Cancer.

[30] Lazova R, Laberge GS, Duvall E, Spoelstra N, Klump V, Sznol M (2013). A Melanoma Brain Metastasis with a Donor-Patient Hybrid Genome following Bone Marrow Transplantation: First Evidence for Fusion in Human Cancer. PLoS One.

[31] Houghton J, Stoicov C, Nomura S, Rogers AB, Carlson J, Li H (2004). Gastric cancer originating from bone marrow-derived cells. Science.

[32] He X, Li B, Shao Y, Zhao N, Hsu Y, Zhang Z (2015). Cell fusion between gastric epithelial cells and mesenchymal stem cells results in epithelial-to-mesenchymal transition and malignant transformation. BMC Cancer.

[33] NatureWang Y, Fan H, Zhou B, Ju Z, Yu L, Guo L (2012). Fusion of human umbilical cord mesenchymal stem cells with esophageal carcinoma cells inhibits the tumorigenicity of esophageal carcinoma cells. Int J Oncol.

[34] Cuyas E, Corominas-Faja B, Menendez JA (2014). The nutritional phenome of EMT-induced cancer stem-like cells. Oncotarget.

[35] Lu X, Kang Y (2011). Cell fusion hypothesis of the cancer stem cell. Adv Exp Med Biol.

[36] Nagler C, Zanker KS, Dittmar T (2011). Cell Fusion, Drug Resistance and Recurrence CSCs. Adv Exp Med Biol.

[37] Dittmar T, Nagler C, Schwitalla S, Reith G, Niggemann B, Zanker KS (2009). Recurrence cancer stem cells--made by cell fusion?. Med Hypotheses.

[38] Fan H, Lu S (2014). Fusion of human bone hemopoietic stem cell with esophageal carcinoma cells didn’t generate esophageal cancer stem cell. Neoplasma.

[39] Berndt B, Zanker KS, Dittmar T (2013). Cell fusion is a potent inducer of aneuploidy and drug resistance in tumor cell/ normal cell hybrids. Crit Rev Oncog.

