# Supplementary figures and images for: Tumorigenic hybrids between mesenchymal stem cells and gastric cancer cells enhanced cancer proliferation, migration and stemness
Source: BMC Cancer. 2015 Oct 24;15:793. doi: 10.1186/s12885-015-1780-1 (PMC4620013; doi:10.1186/s12885-015-1780-1)

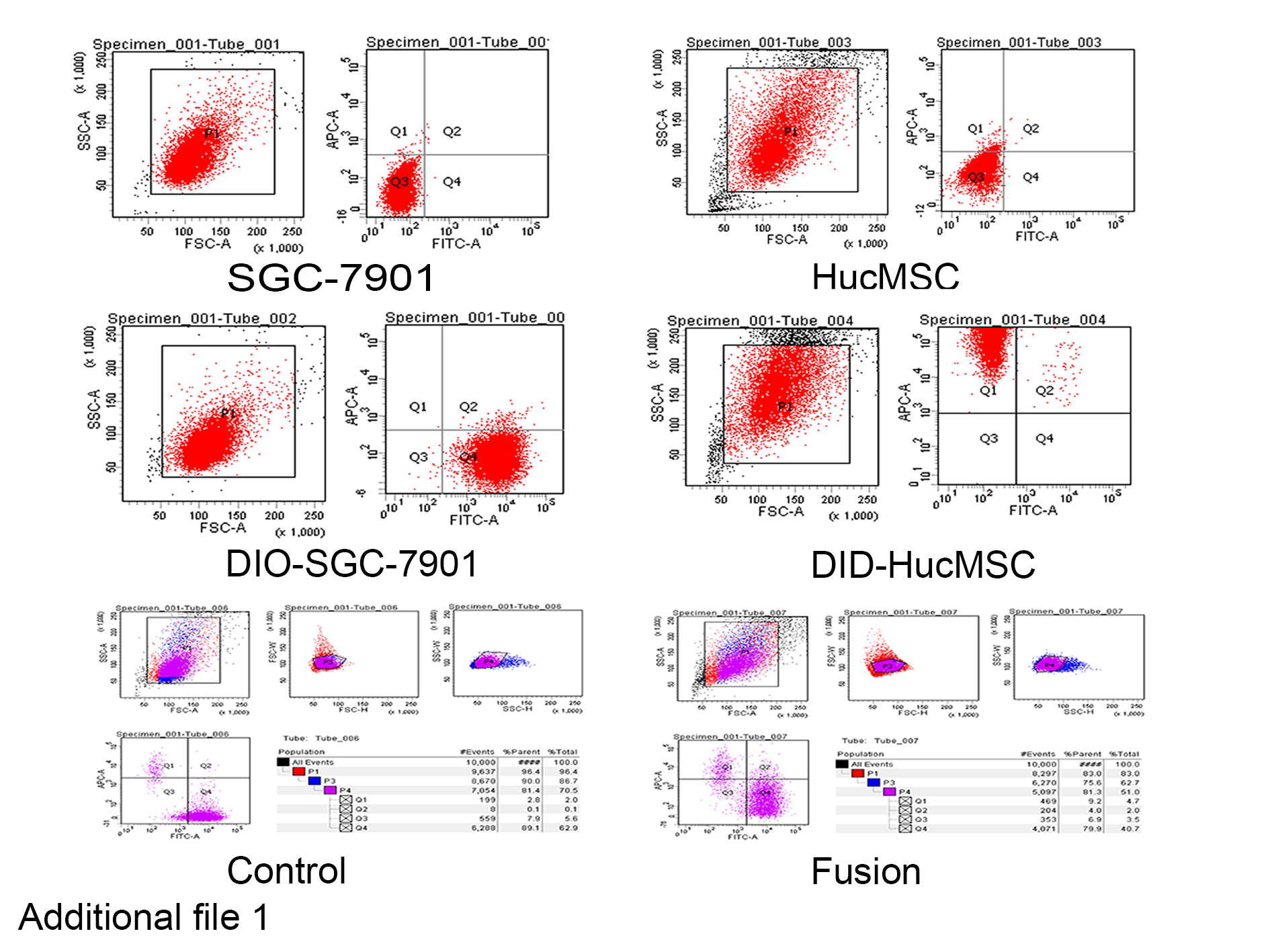

Supplement: Additional file 1: — Flow cytometry analysis and sorting of hybrids. SGC-7901 cells were labeled with DIO, hucMSCs were labeled with DID. The control group was the mock fused stained cells. The double positive fused cells were sorted and collected for the subsequent research. (TIFF 854 kb) [file 12885_2015_1780_MOESM1_ESM.tiff]

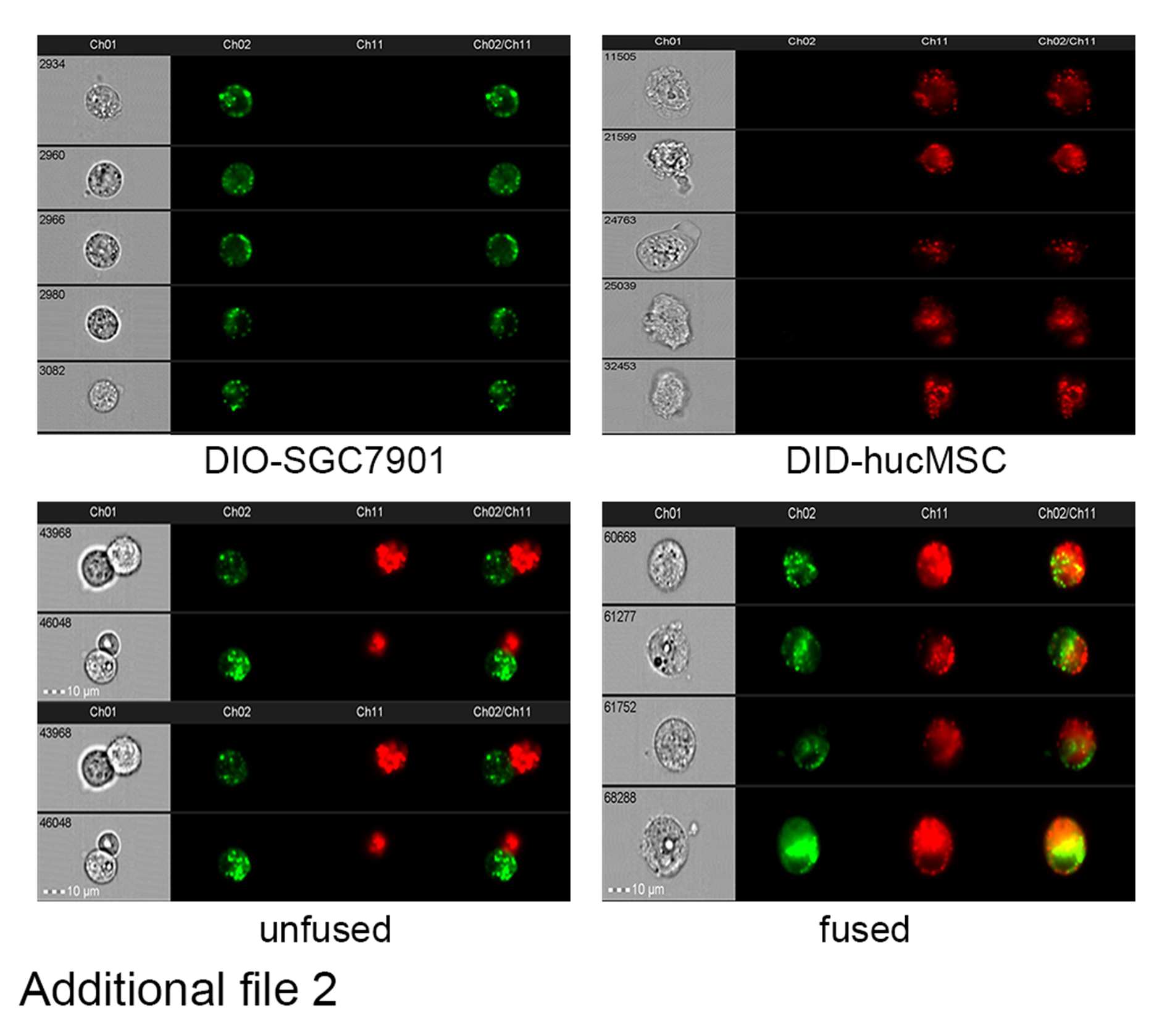

Supplement: Additional file 2: — Representative images from each of the cell population gates. DIO-labeled SGC-7901 showed green, DID-labeled hucMSCs showed red (upper panel). In the fusion cell populations, the unfused cells and fused cells that with double-positive and yellow color were displayed in the lower panel. (TIFF 1104 kb) [file 12885_2015_1780_MOESM2_ESM.tiff]

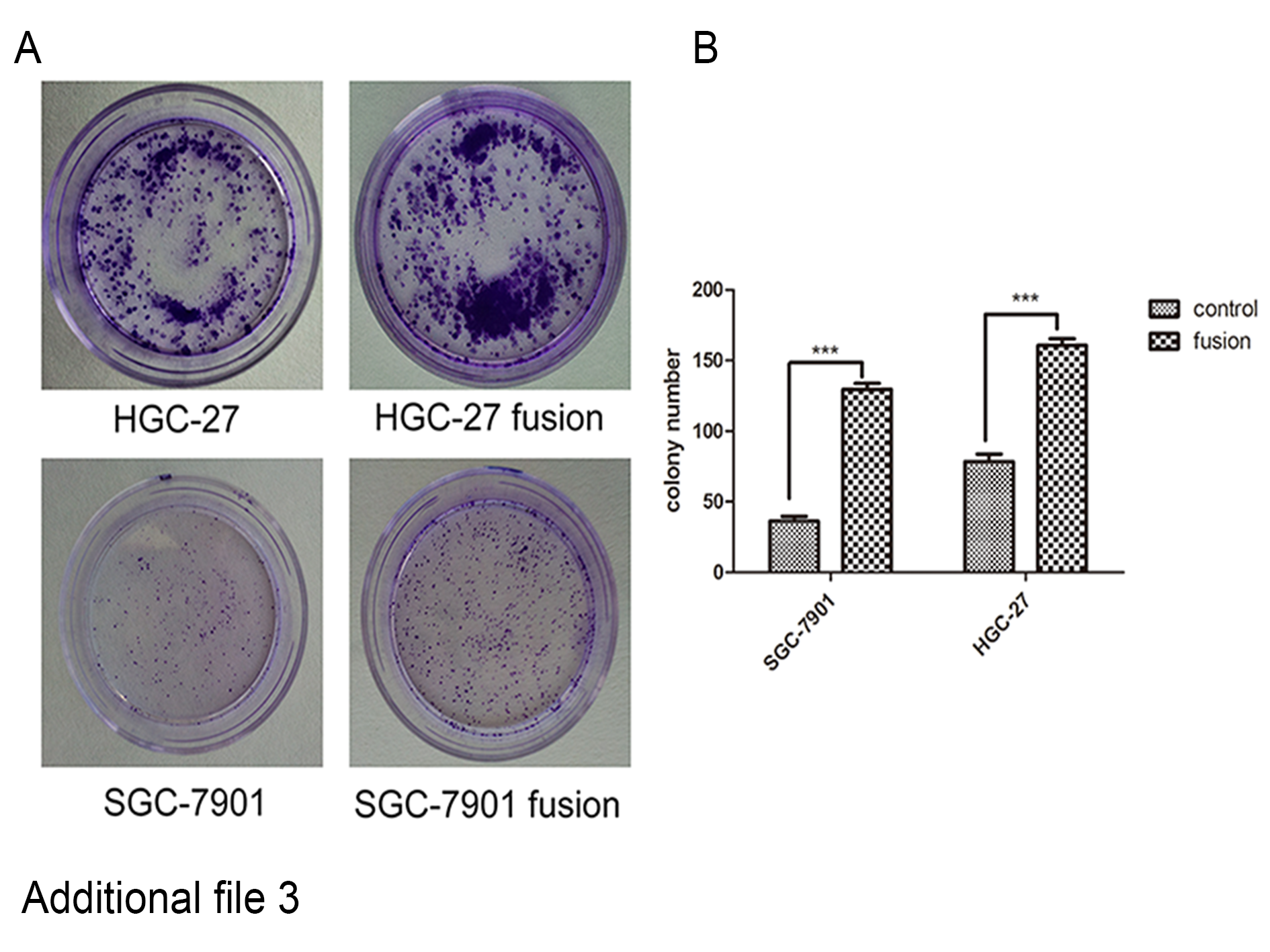

Supplement: Additional file 3: — Representative images of cell colonies for HGC-27 and SGC-7901 parental and hybrid cells. (A) The proliferating ability of the hybrid cells was determined by colony forming assay. (B) Statistical results showed that the hybrid cells grew faster and formed more colonies than parental cells (3–4 folds). (TIFF 1834 kb) [file 12885_2015_1780_MOESM3_ESM.tiff]

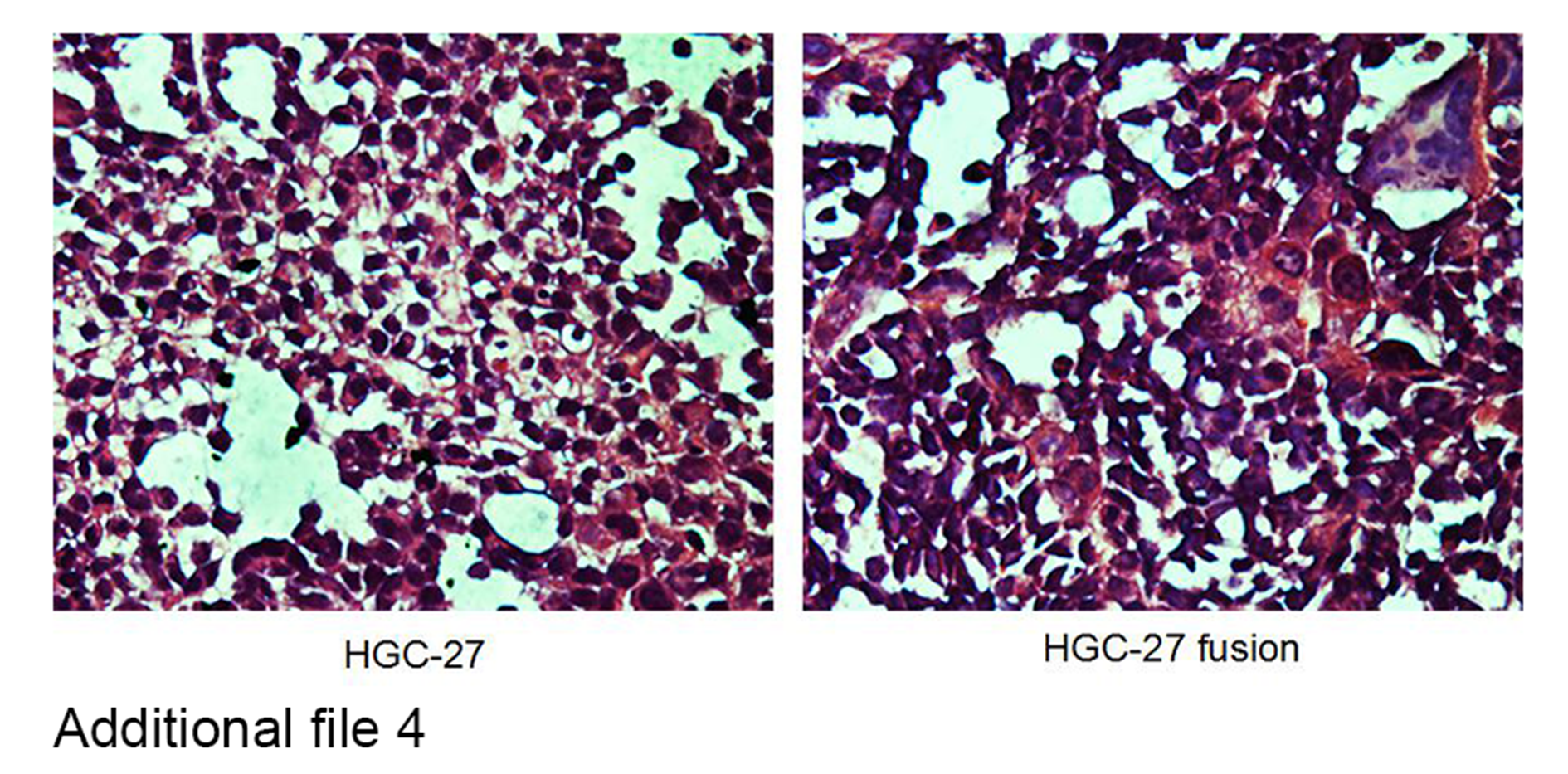

Supplement: Additional file 4: — Histological images of tumor from a mouse injected with the HGC-27 cells and HGC-27 fusion cells. Magnification, ×400. (TIFF 3256 kb) [file 12885_2015_1780_MOESM4_ESM.tiff]
